# On the Role of Fibrocytes and the Extracellular Matrix in the Physiology and Pathophysiology of the Spiral Ligament

**DOI:** 10.3389/fneur.2020.580639

**Published:** 2020-10-27

**Authors:** Noa Peeleman, Dorien Verdoodt, Peter Ponsaerts, Vincent Van Rompaey

**Affiliations:** ^1^Department of Translational Neurosciences, Faculty of Medicine and Health Sciences, University of Antwerp, Antwerp, Belgium; ^2^Laboratory of Experimental Hematology, Vaccine and Infectious Disease Institute (Vaxinfectio), University of Antwerp, Antwerp, Belgium; ^3^Department of Otorhinolaryngology and Head and Neck Surgery, Antwerp University Hospital, Edegem, Belgium

**Keywords:** spiral ligament of the cochlea, cochlea, immune system, sensorineural hearing loss, noise-induced hearing loss

## Abstract

The spiral ligament in the cochlea has been suggested to play a significant role in the pathophysiology of different etiologies of strial hearing loss. Spiral ligament fibrocytes (SLFs), the main cell type in the lateral wall, are crucial in maintaining the endocochlear potential and regulating blood flow. SLF dysfunction can therefore cause cochlear dysfunction and thus hearing impairment. Recent studies have highlighted the role of SLFs in the immune response of the cochlea. In contrast to sensory cells in the inner ear, SLFs (more specifically type III fibrocytes) have also demonstrated the ability to regenerate after different types of trauma such as drug toxicity and noise. SLFs are responsible for producing proteins, such as collagen and cochlin, that create an adequate extracellular matrix to thrive in. Any dysfunction of SLFs or structural changes to the extracellular matrix can significantly impact hearing function. However, SLFs may prove useful in restoring hearing by their potential to regenerate cells in the spiral ligament.

## Introduction

The global prevalence of hearing impairment is increasing significantly. In 1985, around 42 million people suffered from moderate to profound hearing impairment according to the World Health Organization (WHO). This number has risen to ~466 million people in 2018 and is expected to rise to 900 million by 2050 (1). Hearing loss as a consequence of lateral wall dysfunction is often categorized as sensorineural hearing loss. However, it would be more appropriate to refer to this as strial hearing loss, which is caused by both fibrocyte and strial degeneration (2). Due to a lack of sensitive diagnostic testing, the prevalence of strial hearing loss is likely underestimated and therefore often classified as sensorineural hearing loss (3). Hearing loss has a great impact on daily life as it has health (increased rates of hospitalization, higher rates of dementia …), psychosocial and economic effects (4).

## The Auditory System

The auditory system is the body's sensory system responsible for hearing. It consists of three main parts: the outer ear, the middle ear and the inner ear. The outer ear is the external part of the ear and consists of the pinna and the ear canal. It ends at the tympanic membrane, which is the beginning of the middle ear. This contains three ossicles (malleus, incus, and stapes) which are responsible for transferring the vibrations of the tympanic membrane into the inner ear. The inner ear consists of the vestibular apparatus and the cochlea, necessary for balance and sound detection respectively. Defects in the outer and middle ear cause conductive hearing loss, while defects in the inner ear lead to sensorineural hearing loss (SNHL) (5, 6).

### The Cochlea: Anatomy and Histology

**Figure 1:**
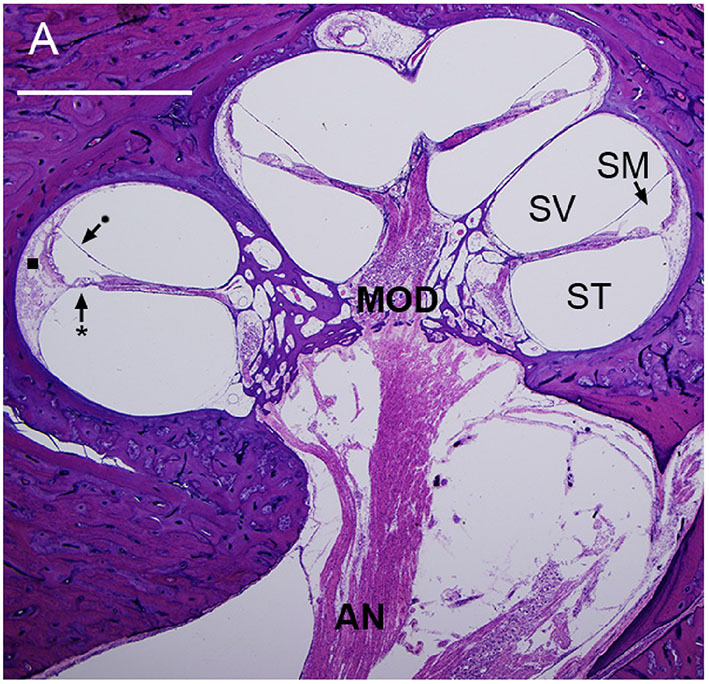
Mid-modiolar section of a human cochlea showing the modiolus (MOD), containing spiral ganglion neurons with axons to and from the auditory nerve (AN). The basilar membrane (*) and Reissner's membrane (•) define three fluid-filled spaces: scala tympani (ST), scala media (SM), and scala vestibuli (SV). The lateral wall (■) connects the basilar membrane to the otic capsule (7).

The cochlea resembles the shape of a snail shell and consists of three fluid-filled canals that run parallel with each other: the scala vestibuli, the scala media, and the scala tympani. The scala vestibuli and tympani contain perilymph while the scala media is filled with endolymph (Figure 1). These two fluids differ in their ionic composition: perilymph resembles extracellular fluid, with a high concentration of sodium, while endolymph resembles intracellular fluid, with a high potassium concentration. The scala media and scala tympani are separated by the basilar membrane. The basilar membrane does not have a uniform thickness which causes different regions to be sensitive to different frequencies. It is widest at the apex of the cochlea and most narrow at the basal region. This causes low-frequency sounds to resonate at the apex, while high-frequency sounds localize at the basal region (8). On top of the basilar membrane sits the organ of Corti, which translates movement of the basilar membrane into electrical impulses. The organ of Corti is also known as the sensory epithelium of the cochlea and consists of a single row of inner hair cells (IHC) and three rows of outer hair cells (OHC) separated by the tunnel of Corti (9). Each hair cell contains stereocilia that deflect with the movement of waves through the endolymph, which will lead to electric impulses that reach the brain *via* the vestibulocochlear nerve (8).

### Histology of the Spiral Limbus

The spiral limbus is situated on the osseous spiral lamina which extends from the modiolus. The spiral limbus mainly consists of fibrocytes which are vulnerable to several stresses such as middle ear infections and acoustic trauma. Interdental cells, located in the top of the limbus, are epithelial cells that anchor the tectorial membrane, a gelatinous structure secreted by the interdental cells that connects to the organ of Corti. It is important for the coupling of sound-induced vibrations to the hair cell stereocilia (10).

### Histology of Lateral Wall Structures

**Figure 2:**
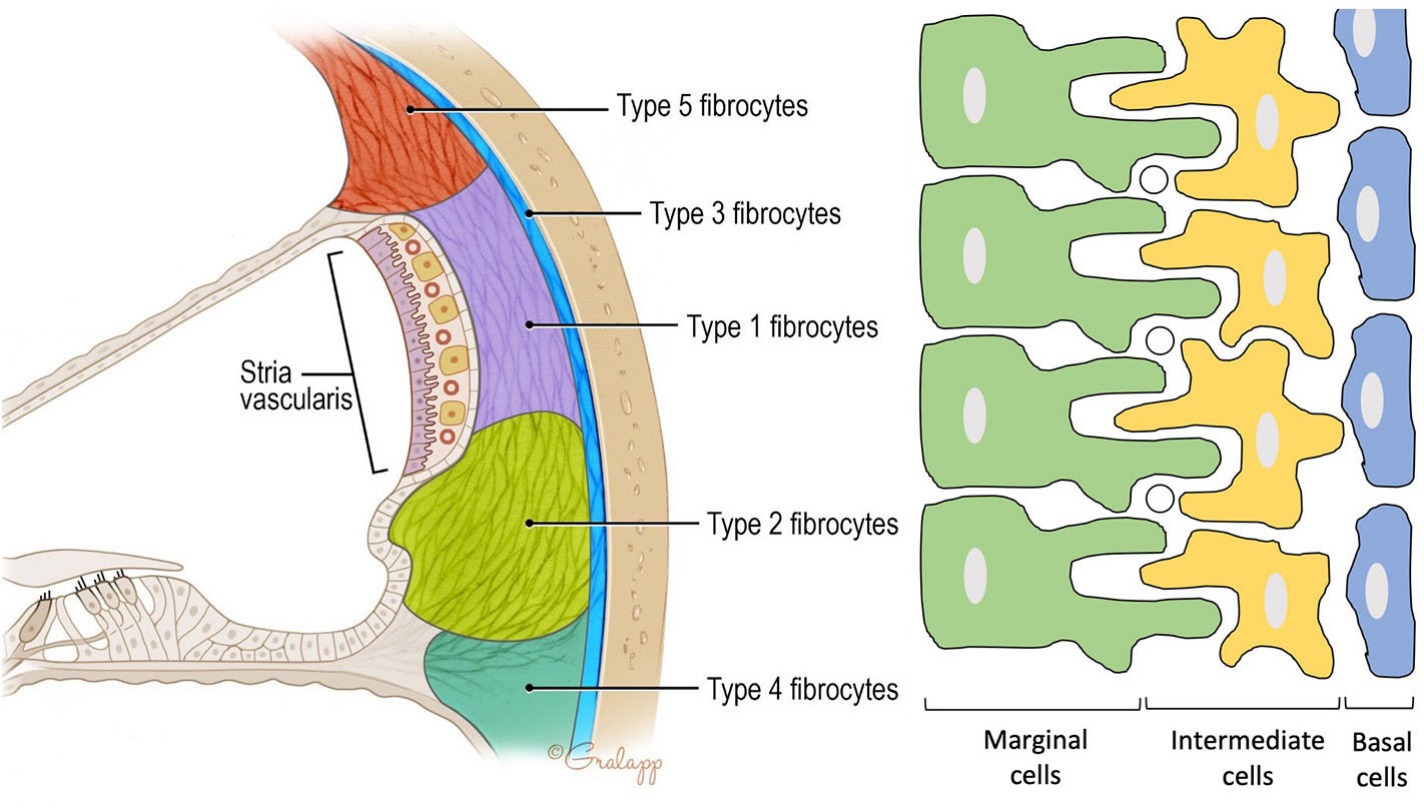
Anatomy of the spiral ligament. (Left) Localization of the different fibrocytes subtypes in the spiral ligament. Cochlin is most abundantly expressed in the region of the type IV fibrocytes. (Right) Schematic representation of the different layers in the stria vascularis consisting of marginal cells, intermediate cells, and basal cells. Marginal and intermediate cells form cell processes. Circles represent blood vessels.

The scala media is lined by the lateral wall (LW) and includes the spiral ligament and the stria vascularis (Figure 2).

#### The Stria Vascularis

Contains three cell types: marginal, intermediate, and basal cells. Marginal cells form the epithelial lining of the scala media. They are the main source of potassium in the endolymph as they accumulate K^+^ ions from the intrastrial space (IS) and export them *via* KCNQ1 channels to the scala media. Intermediate cells are located below the marginal cells and form a network of processes with the marginal cells to maximize the surface for ion exchange. The third cell population are the basal cells which line the lateral surface of the stria. The basal cells are connected to each other by tight junctions that form an ionic barrier between the intrastrial space and the spiral ligament that is filled with perilymph [5, (11)].

#### The Spiral Ligament

Forms the outer wall of the scala media and is connected to the basilar membrane and the Reissner's membrane. It contains blood vessels and five different types of fibrocytes, based on differences in histology and location. Type I and II spiral ligament fibrocytes (SLFs) make up most of the spiral ligament. Type I SLFs are located adjacent to the stria vascularis and are closely associated with tightly packed collagen bundles. Type I SLFs contain relatively few cellular organelles in contrast to type II SLFs. Type II SLFs are located near the spiral prominence between the basilar crest and the stria and play an important role in potassium recycling. Type III SLFs (also called *tension fibroblasts*) are small elongated cells that line the bony otic capsule. Type IV SLFs are small spindle-shaped cells located inferior toward the crista basilaris. Finally, type V SLFs are very similar to type II SLFs and are located at the apical tip, where they are in direct contact with the perilymph of the scala vestibuli. Several ion transporters and proteins are expressed in these SLFs which further differentiate the different types of fibrocytes. These will be discussed in the following section. Numerous intercellular connections are also found which suggest electrical or ionic coupling between the SLFs, important for their functioning (10–13).

## Physiology of Different Cell Types in the Spiral Ligament

SLFs play an important role in ion homeostasis, immune response and regulating blood flow.

### Ion Homeostasis and the EP

One crucial function is the maintenance of an endocochlear potential (EP) of +80 mV in the scale media (14). To enable this, SLFs display a unique ion permeability system. Usually, the resting potential (RMP) of cells is negative under physiological conditions. However, a positive RMP has been observed in SLFs. This is the result of an unusually high permeability to Na^+^, much higher than the permeability to other ions such as K^+^ and Cl^−^. This may play a key role in maintaining a positive RMP of +5 to +12 mV in SLF (15). As a result, the positive RMP contributes to ionic gradients that enable the development of an EP of +80 mV in the endolymph, which is needed for normal hearing function (14).

Multiple ion channels and proteins are expressed on the surface of different types of SLFs, to further increase the RMP (Table 1). Note that there are several other proteins expressed in SLF that are not included in this summary.

**Table 1:** Summary of ion channels and proteins expressed in spiral ligament fibrocytes.

	**Type I**	**Type II**	**Type III**	**Type IV**	**Type V**
Na,K/ATPase	-	+	-	+/-	+
NKCC	-	+	-	+	+
Connexin 26	+	+	-	-	+
Connexin 30	+	+	-	-	+

*NKCC, Na,K-2Cl co-transporter*.

Another factor that contributes to the development of the EP is the potential of the intrastrial space (ISP). The lateral wall can be outlined as a double-layered epithelial system consisting of strial marginal cells and a syncytium composed of SLFs, intermediate and basal cells. In between lies the intrastrial space which has a low K^+^ concentration (16). This is necessary to create a large K^+^ diffusion potential of 90 mV maintained by Na,K/ATPases and NKCCs in the basolateral membrane of marginal cells (17).

Because of this dual system in the LW, the EP can be seen as the sum of two different K^+^ diffusion potentials. First, a diffusion potential that originates from inwardly rectifying K^+^ channels Kir4.1 on the apical membrane of intermediate cells. The second potential comes from KCNQ1 channels apically on the marginal cells. Under physiological conditions, there is a potential difference of 10 mV between the ISP and EP. This is likely generated by K^+^ diffusion as the result of a higher activity of K^+^ in the endolymph compared to the activity of K^+^ inside the marginal cells (16, 18).

**Figure 3:**
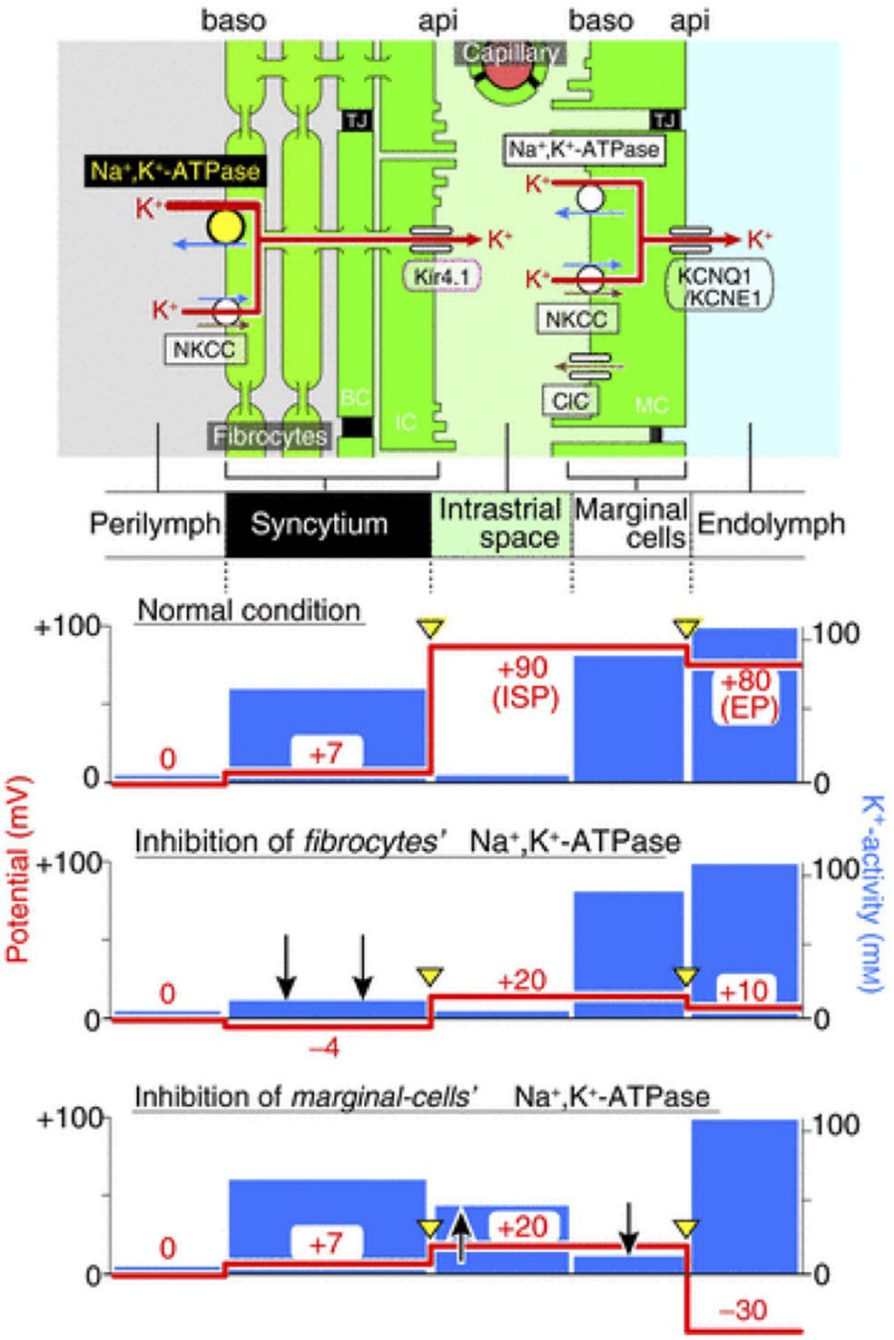
Representation of the electrochemical milieu in the lateral wall (17) Top, scheme of the lateral wall and K+ transport apparatus involved in the generation of the endocochlear potential. The other panels show the predicted potential and K^+^ activity in each compartment under normal circumstances (second panel), during inhibition of Na,K/ATPase of SLFs (third panel) and during inhibition of Na,K/ATPase in marginal cells (fourth panel).

The importance of K^+^ diffusion can be observed when blocking Na,K/ATPases either in the syncytium or in the marginal cells (Figure 3). First, when blocking Na,K/ATPases in the SLFs, the EP drops significantly but remains positive. This is the result of a significant decrease in ISP due to a decrease of K^+^ activity in the syncytium (${\text{aK}}_{\text{syn}}^{+}$). As there is little effect on the activity of K^+^ in the IS (${\text{aK}}_{\text{IS}}^{+}$), the K^+^ gradient greatly diminishes and thereby decreases the K^+^ diffusion potential across the apical surface of the syncytium (17). However, blocking Na,K/ATPases on the basolateral membrane of marginal cells, results in a negative EP. This is due to a decrease in ISP and an increase in the potential difference across the apical membrane of marginal cells (18). For a more detailed description on the molecular and physiological bases of the K^+^ recycling pathways, please refer to Hibino et al. (19).

#### Na,K/ATPase

An important ion channel is the Na,K/ATPase, which is mostly expressed in the type II SLFs, and to some extent in type I and V SLFs (20). It pumps K^+^ ions into the cell and Na^+^ ions out of the cell at the expense of ATP. As this is a form of active transport, a normal function of mitochondria is important to produce sufficient ATP. As a consequence, these SLFs are very susceptible to energy failure, which can cause loss of EP and thus hearing loss. (21)

#### Na,K-2Cl Co-transporter (NKCC)

NKCC is an integral membrane protein consisting of two isoforms. NKCC1 is expressed in many tissues and plays a role in ion homeostasis and cell volume regulation, whereas NKCC2 is expressed solely in the kidney. In the inner ear, NKCC1 is predominantly located in the strial marginal cells and type II and V SLF. A *NKCC1*-knockout mouse model demonstrated a collapse of the lumen of the cochlear duct. Loss of NKCC1 results in a significant decrease of K^+^ secretion and its associated water, which decreases the normal pressure in the perilymph and causes a collapse of several structures and a drop in the EP (22). Type IV SLFs show expression of NKCC1 but not of Na,K/ATPase. This suggests that these cells are subject to osmotic stresses and that NKCC1 is expressed to cope with these stresses to relieve the cells (23). Reduced NKCC1 expression has been observed in aging C57BL/6J mice and may play a role in cellular damage in the stria vascularis and spiral ligament, as a cause of progressive SNHL (24).

#### Connexins

To maintain proper functioning, intercellular communication is critical. This is achieved by connexins (Cx), major players in the formation of gap junctions that allow direct intercellular electrical and metabolic communication. In the inner ear, two separate gap junctional networks have been identified: (1) an epithelial gap junctional network between supporting cells in the sensory epithelium in the organ of Corti, and (2) a connective tissue gap junctional network between connective tissue cells of the lateral wall. Although there are many forms of connexin, Cx26, and Cx30 are the most important connexins in the spiral ligament. Cx26 is mostly located at the apical tip and spiral prominence, where type I, II, and V SLFs are located. On the other hand, Cx30 is located more in the middle area. Type III and type IV SLFs express neither Cx26 nor Cx30, suggesting they are not involved in the gap junctional network and do not play a direct role in K^+^ recycling (25). Mutations in the GjB2 gene encoding Cx26 are the most frequent causes of non-syndromic congenital hearing loss in the Western world (26).

### Immune Response

SLFs play an important role in the inflammatory response toward environmental insults such as bacterial infections, noise trauma, ototoxic drugs, etc. SLFs are able to secrete anti-inflammatory chemokines to protect the cochlea from inflammation leading to hearing impairment (14, 27). Several studies have demonstrated that SLFs secrete monocyte chemoattractant protein-1 (MCP-1), macrophage inflammatory protein-2 (MIP-2), intercellular adhesion molecule-1 (ICAM-1), and vascular endothelial growth factor (VEGF) after stimulation by interleukin 1 (IL-1) and/or tumor necrosis factor α (TNF- α). MCP-1 is a chemoattractant for monocytes and lymphocytes. MIP-2, on the other hand, is a strong chemoattractant for neutrophils (28). TNF-α not only causes SLF to secrete cytokines and chemokines but also plays a role in the activation of Nuclear Factor kappa-light-chain-enhancer of activated B cells (NF-κB). NF-κB is a polymorphic transcription factor that regulates several genes involved in inflammation, cell death, proliferation, etc. The NF-κB pathway is expressed in SLFs but the mechanism of activation and which SLF type expresses NF-κB can differ. Adams et al. reported the highest activation of NF-κB in type II SLFs after administration of lipopolysaccharide (LPS), found on the outer membrane of gram-negative bacteria. However, after noise exposure type I SLFs are the predominant cell type to show NF-κB activation (29).

After acoustic trauma, injured cells may release damage-associated molecular patterns (DAMPs) which are recognized by pattern recognition receptors (PRR) such as Toll-like receptor 4 (TLR-4). Binding of DAMPs to TLR4 recruits and activates cytoplasmic adapter molecules, MyD88 [e.g., (30)]. This results in degradation of I-κBα, an inhibitor of NF-κB, and nuclear translocation of p65 which activates NF-κB and thereby upregulates the transcription of pro-inflammatory cytokines. Type I and II SLFs have demonstrated an increased nuclear translocation of p65 after acoustic trauma, suggesting that NF-κB plays an important role in the regulation of immune responses after noise exposure (31).

### Regulating Cochlear Blood Flow

When an acoustic stimulus activates hair cells, the energy demand in the cochlea increases, which is controlled by the cochlear blood flow (CBF). The CBF is regulated by the end-arterial system of the cochlea (spiral modiolar artery and its arterioles) and by a capillary-based control system. There are two distinct capillary networks in the cochlea. The capillaries of the stria vascularis form the blood-labyrinth barrier, which is crucial for the homeostasis in the cochlea. The second network consists of the capillaries of the spiral ligament, which regulate the CBF. A study by Dai et al., showed evidence of a physical connection between fibrocytes in the suprastrial area (type V SLFs) and capillaries in the spiral ligament through end-foot structures. Moreover, SLFs and vascular cells are also suggested to be coupled by local metabolic signals. Sound stimulation, for example, causes a release of calcium in SLFs and activates the cyclooxygenase (COX) signaling pathway. Consequently, blood vessels are dilated by conversion of arachidonic acid into prostaglandin PGE_2_. Furthermore, COX-1 is selectively expressed in type V SLFs but not in vascular cells, confirming the role of the SLF in CBF regulation (32). Another factor that plays a role in CBF regulation is nitric oxide (NO) (33), which can induce vasodilatation in response to lactate. Lactate is a major product of our metabolism and plays a role in the regulation of blood flow in various organs. In the cochlea, the perilymph contains increased levels of lactate compared to blood and CSF and is upregulated after acoustic stimulation. Extracellular lactate activates neuronal nitric oxide synthase (nNOS) in SLFs through a monocarboxylate transporter, MCT1, and thus stimulates NO production causing vasodilatation (34).

### Glutamate Homeostasis

SLFs are very susceptible to changes in glutamate concentration. The major glutamate transporter of the cochlea is the glutamate-aspartate transporter (GLAST), a high affinity Na^+^-dependent transporter. Glutamate is the major excitatory neurotransmitter between inner hair cells and the spiral ganglion neurons (35). Nonetheless, glutamate uptake is crucial to prevent accumulation which can lead to tonic activation of receptors and thus cause excitotoxicity (36). In mice and several other animal models, GLAST has been identified in the spiral ligament (mainly type II and V SLFs), satellite cells of the spiral ganglia, and supporting cells of the organ of Corti (37, 38). However, a study on human temporal bones was only able to identify GLAST in the spiral ligament and more specifically in type III and IV SLFs (36). GLAST expression in the spiral ligament is necessary to keep the glutamate concentration in the perilymph below damaging levels as the normal concentration of glutamate in the perilymph is low (35). Further relevance of GLAST can be observed in GLAST-deficient mice which demonstrate increased baseline glutamate levels in perilymph and are more susceptible to noise damage (39).

## Physiology of the Extracellular Matrix in the Spiral Ligament

Collagen and cochlin are the most abundant proteins, secreted by SLFs to create a healthy extracellular matrix.

### Collagen

Collagen is the main component of the extracellular matrix in the inner ear. Several types of collagen are expressed but type II collagen (CII) is the most abundant throughout the cochlea and the SL. CII is a fibrillar collagen and the main component of cartilage but is also found in non-cartilaginous tissue such as the cochlea (40). In the spiral ligament, it forms cross-striated, irregularly oriented, thin collagen fibrils which provides stability and strength to the extracellular matrix. It is essential for the integrity of the ion transport systems in the spiral ligament. Furthermore, as it is produced by SLFs, a decrease in CII staining could be a sign of SLF pathology (41, 42). Mutations in *COL2A1*, encoding CII, have been reported to contribute to hearing disorders, such as Stickler syndrome (43). CII has also been proposed to play a role in autoimmune disease associated with hearing loss and vertigo (40). It causes an immune reaction primarily in the tunnel of Corti resulting in a loss of hair cells and degeneration of spiral ganglion cells (44). Other types of collagen such as type V, IX, and XI have also been demonstrated in the inner ear but to a lesser extent in the spiral ligament (45).

### Cochlin

Cochlin, encoded by the *COCH* gene, is a protein that is abundantly expressed in the inner ear, spleen and eye. In the inner ear it is predominantly situated in the spiral ligament and spiral limbus. Next to collagen, it is the most abundant protein in the extracellular matrix of the inner ear. Cochlin is highly conserved among species suggesting that it has crucial functions in cellular processes. However, the exact function of cochlin in the inner ear is not yet fully understood (46, 47).

Its protein structure consists of a signal peptide (SP), a *Limulus* Factor C, Cochlin, and Lgl1 (LCCL/FCH) domain, an intervening domain (ivd1), and two von Willebrand factor A-like domains (vWFA1 and vWFA2), separated by a second intervening domain (ivd2) (48). The vWFA domains show an affinity for specific extracellular matrix components, especially for type I, type II, and type IV collagens. However, the LCCL domain shows no affinity toward these components (49). The LCCL domain has a strong homology to *Limulus* factor C, which is an endotoxin-sensitive serine proteinase involved in the immune response in the horseshoe crab (50, 51).

Previous research has demonstrated that mutations in the *COCH* gene cause DFNA9, an autosomal dominant disorder that causes progressive sensorineural hearing loss associated with vestibular dysfunction (50, 52–55). Mutations can cause misfolding and progressive accumulation of mutant proteins leading to degeneration of dendrites and loss of vestibular and cochlear neurons. However, this explanation does not account for all observed phenotypes as some mutations of cochlin still result in correctly folded protein. A possible explanation for these cases could be the incorrect incorporation of the mutant cochlin in the extracellular matrix of the inner ear (49). Besides DFNA9, cochlin is involved in several other disorders such as Menière's disease and immune-mediated inner ear disease (IMED). IMED is a type of sensorineural hearing loss that causes bilateral hearing impairment and often vestibular symptoms (56). It can occur as an isolated inner ear disease or as an result of a systemic auto-immune disease (57). Cochlin is thought to play an important role in the pathogenesis of IMED, since significantly higher levels of anti-cochlin antibodies have been observed in serum. Furthermore, there are also higher frequencies of IFN-?? producing T-cells and IL-5 producing T cells in response to cochlin (46, 58).

It has been reported that cochlin in the spleen promotes the systemic innate immune reaction against bacterial infection. This is done by cleavage of the LCCL domain from spleen-derived cochlin by aggrecanases and secretion in the blood so the LCCL domain can accumulate in the inflammatory lesions to promote innate immunity (51). Via this mechanism cochlin plays a role in the systemic immune response. In the cochlea, the innate immune response is critical to prevent sensory organ damage to protect the auditory function. Similar to the spleen, the LCCL of cochlea-derived cochlin is cleaved and secreted into the scala tympani to promote an immune response against bacterial infection *via* pathogen segregation. A recent study by Jung et al. demonstrated that *Coch*-knockout mice show less immune response after infection with different bacteria in the scala tympani, incl. *Pseudomonas aeruginosa, Staphylococcus aureus, Haemophilus influenzae, and Streptococcus pneumoniae*, and therefore have more problems clearing the bacterial infection. LCCL acts a chemoattractant of neutrophils and monocytes and recruits them to detect and eliminate the pathogens and promotes cytokine secretion while reducing the bacterial load to the organ of Corti. Furthermore, LCCL appears to directly interact with the bacteria, however, it is still unclear if it binds to LPS or its component, lipid A (50).

## Pathophysiology Related to the Spiral Ligament

### Infection

It has long been thought that the inner ear was an immunologically privileged organ because it is separated from the systemic immune system by the blood-labyrinth barrier and it contains relatively few resident macrophages. However, this hypothesis has been challenged arguing that the cochlea is capable of rapidly recruiting immune cells and therefore inducing an immune response (59). The cochlea contains resident macrophages in the spiral ligament, spiral ganglion, basilar membrane, and stria vascularis. However, they are absent in the scala media and organ of Corti. Macrophages are the major type of immune cells in the cochlea and play several, important roles. They produce pro-inflammatory cytokines and chemokines, participate in immunoregulation, regulate the integrity and permeability of the blood-labyrinth barrier, perform phagocytosis and present antigens. In the basilar membrane, different morphologies can be observed depending on the location. Apically, macrophages show a more ramified and dendritic appearance. While, in the basal part, they have a more amoeboid morphology. After noise exposure, the number of macrophages in the cochlea increases and shows a peak level between 3 and 7 days post exposure (60). This is not due to proliferation of the resident macrophages but due to migration of hematopoietic cells *via* blood vessels in the lateral wall. This migration is caused by secretion of inflammatory mediators by SLFs and other resident cochlear cell types, as mentioned previously. TNF-α plays a major role in the development of cochlear inflammation as it is able to induce the infiltration of inflammatory cells from the systemic circulation into the cochlea even in absence antigens or pathogens. Furthermore, TNF-α is also expressed by leukocytes, which suggests that there is a positive feedback loop to increase recruitment of inflammatory cells. Interestingly, the influx of immune cells is the most significant in the spiral ligament and more precisely, the inferior region where type I and type IV SLFs are situated (59, 61, 62).

Perivascular melanocyte-like macrophages (PVMs) are exclusively found in the stria vascularis and play a role in the immune defense to local noise-induced damage and consecutive repair. PVMs are also responsible for the integrity of the intrastrial fluid-blood barrier which separates the stria vascularis from the peripheral circulation. After acoustic trauma, PVMs can detach from the stria vascularis and cause a significant downregulation of pigment epithelium growth factor (PEGF), which regulates the expression of several tight junction-associated proteins. As a consequence, the permeability of the fluid-blood barrier increases which may lead to influx of toxic substances into the cochlea and a drop in EP (59, 63).

### Noise Trauma

Noise exposure can cause damage to both sensory and non-sensory cells in the inner ear which may lead to decreased hearing function and tinnitus, both reversible and irreversible. Previous research has shown that type IV SLFs are most sensitive to noise exposure and degenerate before hair cells do (11, 64). Noise exposure can result in noise-induced hearing loss (NIHL) by causing threshold shifts, which may be temporary (TTS) or permanent (PTS) (65). Hearing usually recovers within 24–48 h after TTS, but it can last up until 2 weeks. A 94-dB exposure can cause a peak threshold shift of 50 dB at 24 h, which recovered almost completely after 2 weeks. Exposure to 100 dB or more, however, showed irreversible changes (64, 66).

As mentioned previously, ion homeostasis is crucial for maintaining the EP and thus hearing function. However, after intense noise exposure, both the EP and K^+^ concentration in the endolymph decrease dramatically. This decrease can be explained by two mechanisms. First, as the result of an unrestricted movement of K^+^ and Na^+^ between endolymph and perilymph due to uncoupling of the tight junctions through cellular damage in the reticular lamina. Secondly, due to a disrupted ion transport in the stria vascularis and the spiral ligament (67, 68). Noise exposure leads to a prolonged decrease in expression of Cx26 and Cx30 and thereby disrupts the gap junctional intercellular communication (GJIC) (69). A disrupted GJIC can also cause a loss of OHCs and thus decrease hearing function even further (70). Furthermore, Na,K/ATPase activity was also significantly decreased after noise exposure. This decrease could be abolished by both tempol, a free radical-scavenging agent, and a NOS inhibitor suggesting the decrease is either the result of oxidative stress or nitric oxide (69).

A widely used strategy to treat hearing and vestibular disorders is the administration of glucocorticoids (GCs) (71, 72). GCs bind to two types of steroid hormone receptors, glucocorticoid receptors (GC-R) and mineralocorticoid receptors (MC-R). These receptors are normally located in the cytoplasm but after ligand binding, they translocate to the nucleus for activation or repression of target genes. GC are thought to participate in hearing recovery by maintaining ion homeostasis and by their anti-inflammatory and immunosuppressive functions. In the spiral ligament, GC-R are present in all SLF subtypes but most abundant in type III SLFs, whereas MC-R expression is only present in type I and V SLFs (33, 73). Glucocorticoids show a direct effect on Na,K/ATPase expression: increased serum levels of GC are correlated with increased Na,K/ATPase in the lateral wall which suggests that GC regulates the activity of Na,K/ATPase (71). GCs can also control NO production through suppression of inducible nitric oxide synthase (iNOS), which is normally activated after noise exposure in response to TNF-α (33).

#### Histopathology of the Spiral Limbus and Lateral Wall

There are acute (i.e., within 24 h post exposure) and chronic changes visible after noise exposure. In the spiral limbus, degeneration of SLFs can be observed, starting within the apical turn of the cochlea. This degeneration is characterized by nuclear pyknosis and cytoplasmic vacuolation, which are the result of apoptosis. A possible explanation for this loss of SLFs is that noise exposure can lead to insufficiencies of CBF, causing ischemia and damage to the capillaries. This results in an increased NO production that harms the cochlear cells (74).

In the spiral ligament, loss of type IV SLFs can be observed at all levels of noise exposure starting from 94 dB and no signs of regeneration have been observed. In contrast, loss of type II SLFs is only observed at 116 dB in the entire basal turn and there are signs of regeneration after 8 weeks. In acute circumstances, intracytoplasmic vacuoles are observed and in chronic cases, type II SLFs have mostly disappeared from the spiral prominence and the more inferior part of the ligament.

Immediately after noise exposure acute swelling can be observed in the stria vascularis due to increase of the extracellular space between marginal and intermediate cells and swelling of marginal cells. There is also irreversible degeneration of intermediate cells visible. Basal cells that normally form a continuous layer, are more separated and form gaps separating the strial component of the lateral wall from the spiral ligament (11).

An interesting observation is that type III SLFs do not degenerate significantly after noise exposure. In fact, the number of proliferating cells increase in the type III SLF region. These findings suggest that type III SLFs have some type of self-protecting ability. A possible mechanism of this self-protection is that type III SLFs have a strong antioxidant and antiapoptotic function as low immunoreactivity for oxidative stress and apoptosis is observed after trauma (75). Furthermore, type III SLFs are capable of repopulating the type I SLFs region after loss of these cells. One possibility is that because of the rapid proliferation of type III SLFs, they simply replace the type I area. Type III SLFs express aquaporin-1 (AQP1) which is involved in many cell migratory mechanisms, suggesting migration of type III SLFs into the type I area. Another theory is that type III SLFs migrate to the type I SLF area and transdifferentiate in type I SLFs as type III SLFs are considered to have stem cell abilities. Therefore, type III SLFs could play a potential role in regenerative therapies. However, more research is needed to confirm this hypothesis (76).

### Pou3f4

DNF3 is the most common form of X-chromosome linked, non-syndromic hearing loss. It is characterized by conductive hearing loss and progressive sensorineural deafness. Mutations in *Brn-4*/*Pou3f4*, encoding a POU transcription factor, have been identified as the underlying cause (77). During embryonic development, Pou3f4 is expressed in mesenchymal cells of the inner ear but is also associated with neuronal development (78).

*Pou3f4*-knockout mice are profoundly deaf and show an impaired structure of SLFs, whereas the organ of Corti and spiral ganglion appeared normal. Type IV and V SLFs were lost (79), while other SLFs had fewer cytoplasmatic extensions, a reduced volume of cytoplasm and a decrease in number of mitochondria. (77) Furthermore, *Pou3f4*-deficiency also causes disruption of the gap junction plaques (GJPs) resulting in an abnormal morphology of cell-to-cell adhesion of SLFs. Cx26, and Cx30 expression is remarkably reduced and GJPs were significantly shorter, suggesting a degradation of the gap junctional macromolecular complex (78). Expression of Na,K/ATPase and AQP1 was also lost or misplaced in the spiral ligament. In the stria vascularis, there was a decreased cellular integrity and expression of Kir4.1, an inward rectifier K^+^ channel in intermediate cells was lost. As a result of these alterations, the K^+^ transport is severely disrupted leading to a drop in EP and thus hearing impairment (79).

### Mutations in Connexin Genes

*GJB2* or *GJB6*, encoding for connexin 26 and 30, respectively, are two major deafness genes that induce a high incidence of non-syndromic hearing loss, both autosomal dominant (DFNA3) as recessive (DFNB1). However, there is also an association in syndromic hearing loss (80, 81). Mutations in *GJB2* and *GJB6* cause a wide variety of phenotypes resulting in pre- or post-lingual hearing loss ranging from mild to profound deafness (82). A deletion of Cx26 results in cochlear developmental disorders, cell degeneration and reduction of the EP. Cx26 deficiency during the embryonic development leads to attachment of the tectorial membrane to the inner sulcus cells of the organ of Corti and loss of the cochlear tunnel. However, deletion of Cx26 at a later time point resulted in normal cochlear development, suggesting a critical role of Cx26 in the early postnatal development. On the other hand, Cx30 knockout causes increased hearing threshold but results in a normal development of the cochlea and the EP. It causes degeneration of the sensory epithelium postnatally and leads to a decrease in Cx26 expression (83). However, a new Cx30 knockout mouse model with preservation of 50% of Cx26 expression resulted in normal hearing, suggesting that a decrease in Cx26 expression is the main contributing factor in hearing impairment (84) Furthermore, digenic Cx26 and Cx30 heterozygous mutations also lead to hearing loss and a decrease in EP, but no cochlear developmental disorders or cell degeneration (82).

## Recovery of the Spiral Ligament After Trauma

There are three main mechanisms by which the spiral ligament can recover after trauma. Degeneration of SLFs causes impaired K^+^ recycling which decreases the EP. Therefore, it is assumed that reconstruction of the K^+^ pathway by regeneration of SLFs could lead to normalization of the EP and thereby hearing recovery (85). A more general overview of potential therapies in the cochlea against hearing loss can be found elsewhere (86).

First of all, SLFs are able to proliferate and regenerate after trauma. The spontaneous regeneration is mainly the result of mitosis of SLFs around the injured area (87), contrary to spiral ganglion neurons and hair cells, which do not regenerate. However, the self-renewal capacity of SLFs decreases with advanced age (88). Secondly, after trauma has occurred, there is often inflammation and influx of macrophages to the injured region. Several studies have demonstrated that the spiral ligament contains bone marrow-derived cells that can differentiate into macrophages (89). Macrophages are known to promote regeneration after injury (90).

Lastly, the third mechanism has been achieved in experimental conditions and involves the transplantation of mesenchymal stem cells (MSCs). MSCs are multipotent cells that can be isolated from bone marrow (87, 91). The efficacy of this treatment is controlled by the ability of the implanted MSCs to differentiate into SLFs. A study by Kasagi et al. has shown that this efficacy is higher in young mice than in aged mice, which shows that it could be a very promising approach in SNHL in children (91). After transplantation, most of the MSCs can be found in the spiral ligament and differentiated stem cells express several markers typical for SLFs such as Na,K/ATPase and NKCC. However, no trans-differentiation has been observed into spiral ganglion neurons or hair cells (87, 92). Transplanted MSCs also seem to express gap junctions proteins between neighboring cells, such as Cx26 and Cx30, suggesting a reorganization and recovery of the gap junctional network (85). However, validation of the differentiation of MSCs into SLFs was based on immunohistochemistry at a light microscopic level so further validation is still needed for this approach. In addition to differentiating into SLFs, MSCs can also promote regeneration or maintenance of surviving SLFs. MSCs are capable of secreting trophic factors and immunomodulating cytokines and can therefore stimulate regeneration *via* paracrine signaling (93).

## Conclusions

SLFs are crucial in the cochlea as they are involved in ion homeostasis, regulation of cochlear blood flow, immune response, recovery after drug toxicity or noise, and maintaining a healthy extracellular matrix. Any dysfunction of SLFs or structural changes to the extracellular matrix can significantly impact hearing function. However, SLFs may prove useful in restoring hearing by regeneration of cells in the spiral ligament.

## Author Contributions

NP took the lead in writing the manuscript. All authors provided critical feedback, helped shape the research, analysis, and manuscript.

## Conflict of Interest

The authors declare that the research was conducted in the absence of any commercial or financial relationships that could be construed as a potential conflict of interest.

## Abbreviations

CBF: Cochlear blood flow
CII: Type II collagen
COX: Cyclooxygenase
Cx: Connexin
DAMP: Damage-associated molecular pattern
EP: Endocochlear potential
GC: Glucocorticoid
GC-R: Glucocorticoid receptor
GJIC: Gap junctional intercellular communication
GJP: Gap junction plaques
GLAST: Glutamate-aspartate transporter
ICAM-1: Intercellular adhesion molecule-1
IHC: Inner hair cell
IMED: Immune-mediated inner ear disease
iNOS: Inducible nitric oxide synthase
IS: Intrastrial space
ISP: Intrastrial space potential
ivd: Intervening domain
LCCL: *Limulus* Factor C, Cochlin and Lgl1
LPS: Lipopolysaccharide
LW: Lateral wall
MC-R: Mineralocorticoid receptor
MCP-1: Monocyte chemoattractant protein-1
MIP-2: Macrophage inflammatory protein-2
MSC: Mesenchymal stem cell
NF-κB: Nuclear Factor kappa-light-chain-enhancer enhancer of activated B cells
NIHL: Noise-induced hearing loss
NKCC: Na,K-2Cl co-transporter
nNOS: Neuronal nitric oxide synthase
NO: Nitric oxide
OHC: Outer hair cell
PEGF: Pigment epithelium growth factor
PRR: Pattern recognition receptor
PTS: Permanent threshold shift
PVM: Perivascular melanocyte-like macrophage
RMP: Resting membrane potential
SLF: Spiral ligament fibrocyte
SNHL: Sensorineural hearing loss
SP: Signal peptide
TNF-α: Tumor necrosis factor α
TTS: Temporary threshold shift
VEGF: Vascular endothelial growth factor
vWFA: von Willebrand factor A-like.
